# The Effect of CLA-Rich Isomerized Poppy Seed Oil on the Fat Level and Fatty Acid Profile of Cow and Sheep Milk

**DOI:** 10.3390/ani10050912

**Published:** 2020-05-25

**Authors:** Robert Bodkowski, Katarzyna Czyż, Anna Wyrostek, Paulina Cholewińska, Ewa Sokoła-Wysoczańska, Roman Niedziółka

**Affiliations:** 1 Institute of Animal Breeding, Wrocław University of Environmental and Life Sciences, Chełmońskiego 38c, 51-630 Wrocław, Poland; katarzyna.czyz@upwr.edu.pl (K.C.); anna.wyrostek@upwr.edu.pl (A.W.); paulina.cholewinska@upwr.edu.pl (P.C.); 2 The Lumina Cordis Foundation, Szymanowskiego 2a, 51-609 Wrocław, Poland; sokola@libero.it; 3 Institute of Animal Production and Fisheries, Siedlce University of Natural Sciences and Humanities, Prusa 14B, 08-110 Siedlce, Poland; roman.niedziolka@uph.edu.pl

**Keywords:** alkaline isomerization, C18:2 isomers, dairy animals, CLA and TVA, desaturase and atherogenic index

## Abstract

**Simple Summary:**

Conjugated linoleic acid (CLA) has attracted significant interest due to its health-related properties. The use of isomerized poppy-seed oil (IPO) enriched with CLA in cow and sheep feed reduced the fat content in milk and favorably modified the fatty acid profile. The content of saturated fatty acids (SFAs) in milk fat, especially medium-chain fatty acids showing adverse atherogenic and thrombogenic effects, decreased, while the content of polyunsaturated fatty acids (PUFAs), including biologically active fatty acids with pro-health properties (i.e., CLA isomers and *trans*-vaccenic acid (TVA)), increased. In conclusion, IPO with a high concentration of CLA could be used in dairy animal feed to change the nutritional quality and health value of milk, which is beneficial from a human point of view.

**Abstract:**

The aim of the study was to examine the effect of dietary supplementation of isomerized poppy seed oil (IPO) enriched with conjugated dienes of linoleic acid (CLA) on cow and sheep milk parameters (fat content, fatty acid profile, Δ^9^-desaturase index, and atherogenic index). The process of poppy seed oil alkaline isomerization caused the formation of CLA isomers with *cis*-9,*trans*-11, *trans*-10,*cis*-12, and *cis*-11,*trans*-13 configurations in the amounts of 31.2%, 27.6%, and 4.1% of total fatty acids (FAs), respectively. Animal experiments were conducted on 16 Polish Holstein Friesian cows (control (CTRL) and experimental (EXP), *n* = 8/group) and 20 East Friesian Sheep (CTRL and EXP, *n* = 10/group). For four weeks, animals from EXP groups received the addition of IPO in the amount of 1% of dry matter. Milk was collected three times: on days 7, 14, and 30. Diet supplementation with IPO decrease milk fat content (*p* < 0.01). Milk fat from EXP groups had higher levels of polyunsaturated fatty acids, including FAs with beneficial biological properties, that is, CLA and TVA (*p* < 0.01), and lower levels of saturated fatty acids, particularly short- (*p* < 0.01) and medium-chain FAs (*p* < 0.05). The addition of IPO led to a decrease in the atherogenic index.

## 1. Introduction

Fatty acid (FA) composition plays a crucial role in milk nutritional quality. Based on potential benefits for long-term human health, there is interest in developing sustainable nutritional strategies for lowering medium-chain saturated FAs (MCFAs) and increasing specific unsaturated FAs (UFAs) in ruminant milk.

Conjugated linoleic acid (CLA) is a class of FAs comprising as many as 56 isomers with conjugated (neighboring) double bond pairs (i.e., at positions 6,8-; 7,9-; 8,10-; 9,11-; 10,12-; 11,13-; 12,14-; and 13,15- with *cis*-*cis*, *cis*-*trans*, *trans*-*cis*, or *trans*-*trans* geometric configurations) varying along the acyl chain of linoleic acid [1]. Many health-related properties of CLA isomers have been studied, including anticancerogenic, anti-atherosclerotic, antioxidant, and anti-obesity properties, protection of immune system, and contribution to bone formation and body composition [2,3]. There is an extensive literature suggesting that the *trans*-10,*cis*-12 isomer is responsible for a reduction in lipid deposition [4,5] and inhibition of fat synthesis in the mammary gland [6,7].

The most commonly reported recommended CLA intake for humans is 0.8 g/d, but the estimated current average human consumption in Europe, USA, and Canada is 0.21 g/d—ranging from 0.06 g/d in Portugal to 0.40 g/d in Germany—well below the recommended levels. Assuming that the CLA in milk and milk products increases by about 2-fold, the average human consumption would increase from 0.21 to 0.44 g/day [8].

Natural sources of conjugated linoleic acid for the human diet are meat (0.12%–0.68%) and mainly ruminant milk, where they represent 0.34%–1.07% of total FAs [9,10], with the level depending on the species, age, and nutrition [9,11]. Nutritional strategies to increase CLA concentration in dairy products include various systems of feeding [12,13] and supplementation of animal feed with oilseeds or their oils [14,15]. Fish oil is more effective than plant oils for enhancing milk fat CLA content [16,17] and these responses can be further increased when fish oil is fed in combination with supplements rich in linoleic acid (LA) or CLA [18,19]. In the case of commercial preparations containing synthetically or microbiologically produced CLA [6,20], high prices limit their widespread use.

Poppy seed oil exhibited properties similar to those of sunflower oil, was absorbed as well as olive oil, and appeared to be a promising oil for human consumption (rich sources of bioactive compounds, including essential fatty acids, tocopherols, and phytosterols) [21,22]. Poppy seeds are rich in oil, and the oil content amounts to 33%–49% [22,23]. Poppy seed oils contain 86%–90% unsaturated fatty acids, and the most abundant fatty acid is linoleic acid (C18:2) 63%–75% (72% on average), followed by oleic acid (12%–17%) and palmitic acid (8%–10%) [22,23,24]. The oil is a good source of β-sitosterol, campesterol, δ-5-avenasterol, and phenolic compounds and is characterized by high amounts of γ-tocopherol and α-tocopherol [22,25]. According to FAO [26], in 2018, world poppy seed production was 76,240 tonnes, including 47,879 tonnes in Europe, with Turkey being the largest producer (26,991 tonnes), then Czech Republic, India, Australia, France, Spain, Hungary, and China. In the available literature, there is virtually no work on the use of poppy seed oil in animal nutrition, and sparse reports concern, for example, an effect on performance, reproduction, and egg quality parameters [27] and ruminal methane production and fermentation [28].

The aim of the current pilot study was to investigate the influence of addition of isomerized poppy seed oil enriched with conjugated dienes of linoleic acid with *cis*-9,*trans*-11, *trans*-10,*cis*-12, and *cis*-11,*trans*-13 configurations on cow and sheep milk parameters (i.e., fat content, fatty acid profiles, ratios for Δ^9^-desaturase product–substrate, and atherogenic index). The decision to choose poppy seed oil was based primarily on the very favorable profile of fatty acids (high linoleic acid content compared to other vegetable oils) and the search for unconventional oils that can be used in the feeding of ruminants.

## 2. Materials and Methods

### 2.1. Synthesis of CLA Isomers and Feed Additives

The synthesis of CLA isomers from plant oil was conducted according to the methodology elaborated by Walisiewicz-Niedbalska et al. [29], using the process of alkaline isomerization. The raw material was unrefined poppy oil obtained by pressing poppy seeds of the low-morphine cultivar Michałko (IHAR, Plant Breeding and Acclimatization Institute—National Research Institute, Poznań, Poland) in a screw press. The synthesis also involved ethylene glycol (analytical standard, a.s.), potassium hydroxide (a.s.), concentrated hydrochloric acid (a.s.), anhydrous sodium sulfate (a.s.), and hexane as a solvent (all reagents were obtained from Avantor Performance Materials Poland Inc., Gliwice, Poland).

In order to carry out alkaline isomerization of poppy seed oil, ethylene glycol was placed in a reactor, and after heating to about 50–60 °C, approximately 25% mass potassium hydroxide (KOH) relative to glycol was introduced. After dissolving potassium hydroxide and increasing the temperature to 140 °C, poppy oil was added to the system. The isomerization process was carried out for 3 h at 185 ± 5 °C. When the reaction was completed and the reactor content cooled down to 70–80 °C, water was added (1:1 by volume) in order to dilute the resulting soap solution, and then hydrochloric acid was introduced into the reactor in order to acidify the soaps to free FAs. After the soaps were completely acidified, the reaction mixture was transferred to a separator where the aqueous layer was separated, and the FA layer was rinsed with water (about 90 °C) and then dried over sodium sulfate.

Fatty acid methyl esters (FAMEs) were determined using procedures described previously [24]. A chromatographic analysis was performed on the Hewlett Packard 5890 gas chromatograph with a flame-ionization detector (FID), and Supelco SP-2560 capillary GC column (50 m length x 0.25 mm inner diameter (i.d.), d_f_ 0.20 μm; Supelco, Bellefonte, PA, USA). The following oven program was used: 140 °C for 1 min, increase by 1 °C/min up to 180 °C, isotherm for 26 min, increase by 5 °C/min up to 245 °C, and isotherm for 25 min. Other operating conditions are detector temperature, 255 °C; dispenser temperature (split mode), 245 °C; and helium as carrier gas (0.98 mL/min). The identification of common FAs was performed by comparison of sample peak retention times with FAME standards (GLC #47885-U, #46905-U, #CRM46905, and #L8404 from Sigma-Aldrich Chemie GmbH, Schelldorf, Germany; #20-1823-7, #20-1826-7, #20-2013, #10-1875, and #10-1826-90 from Larodan Fine Chemicals AB, Malmö, Sweden). Heptadecanoic acid (#51610; Sigma-Aldrich Chemie GmbH, Schelldorf, Germany) was used as an internal standard. Compositions of FAs in poppy seed oil before (raw form) and after the process of alkaline isomerization are presented in Table 1 (results from 4 analyses, both for raw poppy seed oil and after process of alkaline isomerization, from different batches of oil).

The process of poppy seed oil pressing, its isomerization, and chromatographic analyses of FA profiles were performed at the Ignacy Mościcki Institute of Industrial Chemistry in Warsaw (Poland).

Isomerized poppy seed oil (IPO) was nozzle-sprayed onto Humokarbowit^®^. The spraying process (20% IPO/kg of carrier) was performed by the Tronina company (PHW Tronina, Raków, Poland).

### 2.2. Animals and Treatments

All the cows and sheep were handled in accordance with the regulation of the Polish Council on Animal Care, and all procedures for this trial were approved by the 2nd Local Ethical Committee for Experiments on Animals in Wrocław (No. 6/2009).

The study was carried on sixteen Polish Holstein Friesian cows of black-white variety (582 ± 12.1 kg of BW, at 90 ± 12 d of lactation (multiparous cows), and an average milk yield of 31.4 ± 3.57 kg/d), divided into two groups (*n* = 8/group). The animals were selected on the basis of stage of lactation, milk yield, and live weight and they were allocated at random to particular groups. The cows were kept in a stall system, individually blocked, and fed total mixed ration (TMR) based mainly on maize and grass silage with free access to fresh water. Cows from the experimental group (EXP) additionally received isomerized poppy seed oil (IPO) in the amount of 1% dry matter (DM), that is, 220 g per head/day on the mineral carrier Humokarbowit, while the animals from the control group (CTRL) received Humokarbowit in an analogous amount (Table 2). Dry matter intake (DMI) in both groups was 21 kg/cow per day, allowing 5% refusals.

In the case of sheep, the research material consisted of twenty Friesian breed ewes (62 ± 2.6 kg of BW, at 110 ± 12 d of lactation (multiparous sheep in 3rd–4th lactation), and an average milk yield of 1.96 ± 0.34 kg), divided into two groups (*n* = 10/group). The sheep were kept individually indoors and fed grass hay and complex mixture (62%:38% of DM) with free access to fresh water. Ewes from the experimental group (EXP) additionally received isomerized poppy seed oil (IPO) in the amount of 1% DM, that is, 20 g per head/day on the mineral carrier Humokarbowit, while sheep from the control group (CTRL) were given Humokarbowit in the same amount (Table 3). In both groups, DMI was 1.9 kg/ewes per day, allowing 7% refusals.

Animals (cows and ewes) with similar body weight and milk yield (no statistical differences) were selected for the study and the allocation to particular groups (CTRL and EXP) was random.

Feed doses for cows and sheep were formulated according to IZ PIB-INRA [30] using INRAtion 4.0 software (DJ Group, Cracow, Poland), based on chemical analyses of ingredients according to AOAC [31] procedures: dry matter (method 934.01), crude protein (method 984.13; Kjeltec™ 2300 apparatus; FOSS Analytical AB, Höganäs, Sweden), crude fat (method 920.39), and crude fiber (method 978.10; Fibertec™ 1020 (M6) apparatus; FOSS, Apeldoorn, Netherlands) (Table 2 and Table 3).

### 2.3. Experiment Design, Measurements, and Sampling Procedures

Before the start of the experiment, all cows and sheep were fed the same CTRL diet for 10 days (considered the adaptation period; Table 2 and Table 3). The study was conducted for a 4-week period. The supplements (Humokarbowit in CTRL and Humokarbowit with isomerized poppy seed oil in EXP) were administered in the morning for 30 d (cows at 05:00 h with TMR and sheep at 07:00 h with complex mixture).

The animals were milked twice a day (cows at 06:00 and 16:00 h, sheep at 08:00 and 18:00 h). Individual milk samples (100 mL each) from the morning milking were collected from cows and sheep after 7, 14, and 30 days for analysis of fat content and FA profiles. Milk samples were immediately cooled to a temperature of 4 °C; transferred to the Laboratory of Milk Assessment and Analysis in the Institute of Animal Breeding, Wrocław University of Environmental and Life Sciences (Poland); and analyzed within 4 h of collection. Milk fat content was determined by automated infrared (Infrared 150 apparatus; Bentley Instruments Inc., Chaska, MN, USA) and is presented in Table 4a and Table 5a.

Milk fat (1 g) for chromatographic analysis was obtained after milk centrifugation at 4000 × *g* for 15 min at 4 °C (MPW 260RH apparatus; Med. Instruments, Warsaw, Poland). Lipid extraction was performed according to the modified Folch procedure (chloroform and methanol in a volume ratio of 2:1), and methylation was performed using 2 M KOH in methanol [19].

The chromatographic analyses were conducted using capillary gas chromatography under the following conditions: Agilent Technologies 7890A gas chromatograph with an FID detector, and HP-88 capillary GC column (100 m length × 0.25 mm i.d., d_f_ = 0.25 μm; Agilent Technologies, Santa Clara, CA, USA). The following temperature program was used: initial temperature 100 °C (5 min), increase by 4 °C/min up to 140 °C, increase by 2 °C/min up to 240 °C, and final isotherm for 5 min. Injection was at 1 μL in split mode (80:1 split ratio), injector temperature was 250 °C, detector temperature was 270 °C, and helium was used as the carrier gas. FAME standards (GLC #47885-U, #46905-U, #CRM46905, and #L8404 from Sigma-Aldrich Chemie GmbH, Schelldorf, Germany; #20-1823-7, #20-1826-7, #20-1822, #20-2013, #10-1876-0, #10-1875, #30-1826-90-0, and #30-1826 from Larodan Fine Chemicals AB, Malmö, Sweden) were used to identify FAs with the Agilent ChemStation software (Agilent Technologies, Santa Clara, CA, USA). The composition of FAs in cow and sheep milk fat is presented in Table 4a and Table 5a.

Chromatographic analyses in this part of the study were conducted in the Laboratory of Chromatography and Meat Analysis, Institute of Animal Breeding, Wrocław University of Environmental and Life Sciences (Poland).

The desaturase index (DI) was calculated for the 4 pairs of fatty acids that represent products and substrates for Δ^9^-desaturase, defined as (product of Δ^9^-desaturase)/(substrate of Δ^9^-desaturase + product of Δ^9^-desaturase). The atherogenic index was calculated as the content ratio of (C12 + (4 × C14) + C16)/(MUFAs + PUFAs) proposed by Ulbricht and Southgate [32].

### 2.4. Statistical Analysis

All variables were tested for normality using the Shapiro–Wilk test. Experimental data were analyzed using SAS (Statistical Analysis System, version 9.3 for Windows; SAS Institute, Cary, NC, USA). The fat content and composition and fatty acid profile of cow and sheep milk were calculated by two-way ANOVA with dietary treatments (D) and sampling time (T) as fixed effects and their interactions (D × T) according to the model:

*Y_ijk_* = *μ* + *D_i_* + *T_j_* + *D_i_* × *T_ij_* + *ε_ijk_*

where *Y_ijk_* is the dependent variable, *μ* is the overall mean, *D_i_* is the effect of dietary treatment (*i* = CTRL from days 7, 14, and 30; EXP from days 7, 14, and 30), *T_j_* is the effect of sampling time (*j* = CTRL and EXP from day 7, CTRL and EXP from day 14, and CTRL and EXP from day 30), *D* × *T_ij_* is the interaction between dietary treatments and sampling time, and *ε_ijk_* is the residual error.

Differences in ANOVA were determined using Tukey’s multiple range test at *p* ≤ 0.05 levels of significance.

The data are presented as average values and accompanied by standard error of the mean.

## 3. Results

### 3.1. Synthesis of CLA Isomers

As a result of the cold-pressing process, 53% of low-morphine oil was obtained from poppy seeds, characterized by a high content of linoleic acid (i.e., about 73%) (Table 1). Similar to other unprocessed vegetable oils, raw poppy seed oil did not contain CLA. As a result of the alkaline isomerization process, CLA isomers of *cis*-9,*trans*-11 (c9,t11), *trans*-10,*cis*-12 (t10,c12), and *cis*-11,*trans*-13 (c11,t13) configurations were synthesized in the amounts of 31.2%, 27.6%, and 4.1% of total FAs, respectively (Table 1). Isomerized poppy seed oil (IPO) also included saturated fatty acids (SFAs) with chain lengths of C16–18 (14.2%) and unsaturated fatty acids (UFAs): 12.8% oleic acid (OA, C18:1 c9), 6.8% linoleic acid (LA, C18:2 c9,c12), 0.3% α-linolenic acid (ALA, C18:3 c9,c12,c15), and 0.2% conjugated α-linolenic acid C18:3 isomers (CLnA) (Table 1).

### 3.2. Milk Fat Content and Fatty Acid Profiles

As a result of supplementation of dairy cows’ TMR with IPO, a decrease in milk fat content after 7 days by 13.1% (*p* < 0.01), after 14 days by 16.8% (*p* < 0.01), and after 30 days by 20.2% (*p* < 0.01) was observed compared with the CTRL group (Table 4a). A decrease in milk fat content, as a result of IPO additive administration, was also found in sheep milk: 11.5% (7 d, *p* < 0.05), 20.2% (14 d, *p* < 0.01), and 24.4% (30 d, *p* < 0.01). In both groups of animals, in addition to the diet, the fat content of milk was also affected by the duration of IPO supplementation (*p* < 0.05) and a diet x time interaction was recorded (*p* < 0.01) (Table 5a).

The addition of IPO also modified FA profiles of milk fat and affected the value of atherogenic index and the product/substrate ratio for Δ^9^-desaturase (Table 4a,b and Table 5a,b).

In cows, the proportion of total SFAs in milk fat decreased by 4.6% (*p* < 0.05), 6.2% (*p* < 0.01), and 4.7% (*p* < 0.05) during all the milking days, that is, on days 7, 14, and 30, respectively, compared with the CTRL group (Table 4b). In the group of SFAs, there was a decrease in the proportion of short-chain FAs (SCFAs, C4–10:0) by 30.3%, 33.8%, and 32.5% (all differences at *p* < 0.01), respectively, after 7, 14, and 30 days as well as in the proportion of medium-chain FAs (MCFAs, C12–16), that is, laurynic acid (C12:0) from 7.4% (7 d, *p* < 0.05) to 16.6% (30 d, *p* < 0.01), myristic acid (C14:0) from 4.9% (7 d) to 12% (14 d, *p* < 0.05), and palmitic acid (C16:0) from 4% (7 d) to 6.6% (14 d, *p* < 0.05) (Table 4a,b). The atherogenic index (AI) of milk decreased by 10.6% (after 7 days, *p* < 0.01) to 20.5% (after 14 days, *p* < 0.01), as did the desaturase index (DI) of C18:1/C18:0 and C18:2 c9,t11/C18:1 t11 (Table 4b). During all milking days (i.e., on the 7th, 14th, and 30th days of the experiment), as a result of supplementation of cows’ diets with IPO addition, increases were observed for the content of *trans*-vaccenic acid (TVA) (123.3%, 164.1%, and 142.2%, respectively), CLA isomers of configuration c9,t11 (92.6%, 117.3%, and 122.4%, respectively) and t10,c12 (100%, 75%, and 100%, respectively) (all differences were statistically significant at *p* < 0.01), and stearic acid C18:0 (17.9%, *p* < 0.05; 23.9%, *p* < 0.01; and 29.1%, *p* < 0.01, respectively) compared with the CTRL group (Table 4a). The addition of IPO also increased monounsaturated fatty acid (MUFA) content in milk fat after 7, 14, and 30 days of application by 4.3%, 12.6% (*p* < 0.01), and 10.1% (*p* < 0.05), respectively. There were also increases in polyunsaturated fatty acids (PUFAs) by 20%, 18.4%, and 19.9% and in total CLA by 93.2%, 113.8%, and 122.2%, respectively, after 7, 14, and 30 days (all differences were statistically significant at *p* < 0.01) (Table 4b). In addition to the described dietary effect, the time of the IPO supplementation also influenced the content of C4, C10, C12, C14, C18, TVA, C18:2 t10,c12 (CLA), SCFAs, and desaturase index CLA/TVA (*p* < 0.05, *p* < 0.01). In the group of animals receiving the addition of IPO, milk fat on the 14th day of the experiment had a statistically lower SCFA and C10:0 content compared with that from the 7th day (*p* < 0.01). Moreover, a diet x time interaction was noted (*p* < 0.05, *p* < 0.01) in the case of C4, C6, C10, C12, C16, C18, CLA isomers, SCFAs, MCFAs, PUFAs, Σ CLA, desaturase index CLA/TVA, and AI (Table 4a,b).

A similar spectrum of changes in FA profiles was also observed in sheep milk fat. During the whole experiment, the share of SCFAs (C4–10) decreased by 29.6%, 36.2%, and 41.7% (all differences were statistically significant at *p* < 0.01), respectively, after 7, 14, and 30 days compared with the CTRL group (Table 5b). A decrease in the share of MCFAs (laurynic acid by 18.8% (after 7 d, *p* < 0.01) to 29.6% (after 14 d, *p* < 0.01) and myristic acid by 5.5% (after 30 d, *p* < 0.05) to 9.4% (after 7 d, *p* < 0.01)) and a decrease in palmitic acid and total content of SFA level were also observed (*p* < 0.05) (Table 5a,b). The value of milk AI also decreased by 15.7% (7 d, *p* < 0.01) to 19.7% (30 d, *p* < 0.01) as did the DI of oleic acid to stearic acid and rumenic acid (RA) to TVA (*p* < 0.01) (Table 5b). As a result of ewes’ feed dose supplementation with IPO, the proportion of stearic acid C18:0 in milk fat increased by 33.3%, 29.7%, and 41%; TVA by 145.3%, 161.3%, and 199.2%; CLA isomers with c9,t11 configuration by 83.1%, 92.3%, and 119%; and CLA isomers with t10,c12 configuration by 166.7%, 133.3%, and 266.7%, respectively, after 7, 14, and 30 days (all differences at *p* < 0.01) (Table 5a). After 7, 14, and 30 days of IPO application, an increase in the proportion of MUFAs was also observed in milk fat: 6.3% (*p* < 0.05), 8.9% (*p* < 0.01), and 10% (*p* < 0.05), respectively. PUFAs increased by 15.9%, 34.8%, and 29.7% and total CLA increased by 85.9%, 94.3%, and 125%, respectively, after 7, 14, and 30 days of IPO application (all differences at *p* < 0.01) (Table 5b). Based on the statistical analysis, it was found that the time of IPO application affected the content of C4, C6, C8, C10, C12, C16, C18, TVA, CLA isomers, Σ CLA, PUFAs, long-chain polyunsaturated fatty acids (LC-PUFAs), SCFAs, desaturase index C18:1/C18, and AI (*p* < 0.05, *p* < 0.01). In the group of animals receiving the addition of IPO, milk fat on the 30th day of the experiment was characterized by higher content of total CLA, CLA isomers c9,t11 and t10c12, and TVA and lower content of C4:0 and C6:0 compared with milk fat on the 7th day of the experiment (*p* < 0.01). Diet x time interaction was noted for C4, C6, C8, C10, C14, C16, C18, C18:1 t10, TVA, C18:2, all CLA isomers, Σ CLA, PUFAs, MUFAs, SCFAs, desaturase index C18:1/C18, and AI (*p* < 0.05, *p* < 0.01) (Table 5a,b).

## 4. Discussion

### 4.1. CLA Synthesis and Elaboration of Feed Additives

The content of CLA synthesized as a result of the alkaline isomerization process is significantly affected by the FA profile of the substrate. A high content of LA, which is the main substrate in the process of CLA synthesis, and a low content of ALA, which is also subject to isomerization leading to the formation of positional and geometric isomers (CLnA), determine the high degree of usefulness of the oil.

From the point of view of CLA isomer synthesis, poppy seed oil containing 72.8% LA and 0.7% ALA (Table 1) is characterized by a very favorable FA profile. Similar to other unprocessed oils of plant origin, this does not contain CLA. The distribution of double bonds (positional) and the arrangement of radicals in relation to the double bond axis (geometric) in LA change as a result of the alkaline isomerization process. Consequently, three conjugated dienes of linoleic acid with c9,t11, t10,c12, and c11,t13 configurations, having biological properties other than LA, in amounts of 31.2%, 27.6%, and 4.1% of total FAs, respectively, were synthesized. Apart from CLA, the IPO also included SFAs (C16–18:0), OA, LA, ALA, and CLnA (Table 1).

In the pilot studies, we decided to use poppy seed oil because of its very beneficial FA profile in terms of CLA synthesis. Favorable FA profiles from the point of view of CLA synthesis by the process of alkaline isomerization have also been characterized, for example, in grape seed oil (68.2%–70.1% LA, 0.4% ALA), sunflower oil (57.5%–61% LA, 0.9% ALA), soybean oil (40%–47% LA, 5.5%–7% ALA), primrose oil (69.1% LA), blackcurrant seed oil (46.3% LA), and milk thistle oil (57.1% LA, 0.1% ALA) [33].

Due to the oily form of IPO, in order to facilitate its use in animal feed, it was nozzle-sprayed on the mineral carrier, Humokarbowit^®^, a humus-mineral preparation characterized by high sorption capacity and antioxidant properties. The composition of Humokarbowit® was previously described by Bodkowski and Patkowska-Sokoła [34], and it was demonstrated to be suitable for farm animal feed [4,19].

### 4.2. Fat Content

According to literature data, certain types of diet may cause a significant decrease in milk fat secretion. Low-fat milk syndrome, more commonly referred to as milk fat depression (MFD), is a state that naturally occurs in dairy production when animals are fed highly fermentable diets or dietary plant or fish oil supplements [35,36]. As a result of disturbances in the rumen biohydrogenation process, FAs are produced as intermediate products, which are strong inhibitors of milk fat synthesis [37,38]. The MFD mechanism focuses on the mammary gland and includes a coordinated reduction of mRNA genes of key enzymes associated with all aspects of milk fat synthesis [38,39]. These processes include de novo synthesis of FAs, collection and transport of previously produced FAs, and denaturation and incorporation into triglycerides (TGs). The *trans*-10,*cis*-12 CLA isomer has been proven to be a potential nutritional tool to manipulate milk fat synthesis and FA profiles in lactating animals [17,38,40]. The activity of desaturase Δ^9^, responsible for fat synthesis in the mammary gland, was probably also inhibited by TVA formed from CLA during ruminal transformations [41].

Baumgart et al. [42] indicated that abomasal infusion of 3.5, 7, and 14 g/d of *trans*-10,*cis*-12 CLA in cows for five days decreased milk fat concentration by 24%, 37%, and 46%, respectively, while both isomers *trans*-10,*cis*-12 and *cis*-9,*trans*-11 CLA decreased milk fat concentration by 28%, 40.8%, and 38.6%–58.1%, respectively, in the studies by Urrutia and Harvatine [20], Vyas et al. [43], and Haubold et al. [7]. In the experiment by Dohme-Meier and Bee [44], the supplementation of unprotected CLA decreased fat content in cow milk by 53.8%. In the experiment by Kowalski et al. [45], supplementation with the rumen-protected form decreased fat content by 10.2%. In a study of ewes, supplementation with 9 g/d of t10,c12 CLA decreased the milk fat content by 31.3% [46] and 27 g/d of CLA (29.9% t10,c12) reduced milk fat concentration by 28%, 26%, and 42% during early, mild, and late lactation, respectively [47]. The doses of 10, 20, and 30 g/d of rumen unprotected CLA (containing 29.9% of t10,c12) inhibited milk fat synthesis by 6.5%, 14.2%, and 25.5%, respectively [48]. The effect of the lowering of milk fat content by 18% and 22.3% induced by t10,c12 CLA was also demonstrated by Toral et. al. [6] and Sandri et al. [49], respectively.

The antilipogenic effect of *trans*-10,*cis*-12 CLA was also observed in lactating goats [50,51], but they were apparently less responsive than cows and ewes [7,52].

In the present study, the IPO supplement contained two major CLA isomers: *trans*-10,*cis*-12 and *cis*-9,*trans*-11. In previous studies, Baumgard et al. [53] and Bauman et al. [54] had shown that the c9,t11 CLA isomer had no or little effect on milk fat synthesis in dairy cows; hence, the lowering effect of fat can be attributed to the t10,c12 CLA isomer. In our study, the effective amount of t10,c12 isomer was 60.7 and 5.5 g/head/day in cows and sheep, respectively, and the decrease in milk fat concentration was from 13.1% to 20.1% in cows and from 11.5% to 24.4% in sheep, depending on the duration of IPO application.

### 4.3. Fatty Acid Profiles

Milk fat synthesis depends on two general sources of FAs, namely, de novo synthesis of FAs in the mammary gland and transfer of preformed FAs from blood TGs. The short- and medium-chain FAs (SMCFAs, C4–C14) and half of C16 FAs are synthesized de novo, whereas the rest of the FAs including C16 and other long-chain FAs (LCFAs) are derived from TGs in the blood or from non-esterified fatty acids (NEFAs), mainly during negative energy balance [54]. About half of the fat in milk is synthesized in the udder from acetate and β-hydroxybutyrate and the remaining part is transported from the pool of FAs circulating in the blood (originating from body fat mobilization, absorption from diet, or from fat metabolized in the liver) [55]. During diet-induced MFD, the de novo synthesized SMCFAs are reduced to a much greater extent than the other saturated FAs [6,18,56]. An effect of CLA isomers on lipid metabolism and decrease in the short- and medium-chain FA (SMCFA) level in milk is confirmed in the studies by Bauman et al. [54] and Harvatine et al. [57], and this is probably related to the activity of many lipogenic enzymes, such as acetyl-CoA carboxylase (ACC) and fatty acid synthase (FAS) [58], and decreases lipogenic rates and expression of genes involved in milk lipid synthesis [56].

In our study, the concentrations of all individual SCFAs, including C4, C6, C8, and C10 (*p* < 0.01), and MCFAs, including C12, C14, and C16 (*p* < 0.01, *p* < 0.05), were significantly reduced as the result of cow and sheep diet supplementation with IPO, while the concentration of C18 (*p* < 0.01) was increased. In the case of cows, except the diet, the content of C4, C10, C12, and C14 was also affected by the duration of the IPO supplementation (*p* < 0.05, *p* < 0.01); and for C4, C10 (*p* < 0.01) as well as C6, C12, and C16 (*p* < 0.05), the diet x time interaction was also observed. On the other hand, in the case of sheep, the IPO supplementation time affected the content of C4, C6, C8, C10, C12, and C16 (*p* < 0.05, *p* < 0.01), while diet x time interaction was recorded for C4 and C6 (*p* < 0.01) as well as C8, C10, C14, and C16 (*p* < 0.05). Similar changes in FA profiles in milk, as the result of infusion or supplementation of CLA isomers and their mixture, have been demonstrated in other studies on cows [43,44,59] and sheep [46,52]. The decrease in the share of MCFAs in milk fat (i.e., laurynic, myristic, and palmitic acids) and the unquestionable role of total cholesterol and its LDL fractions in blood as dietary factors for the cause of many cardiovascular diseases [32,60] are very important observations from previously conducted research.

On the other hand, the content of biologically active FAs with pro-health properties [2,61] (i.e., isomers c9,t11 and t10,c12 CLA and TVA, as well as PUFAs and MUFAs) significantly increased as a result of IPO application in milk fat of cows and sheep. In both groups of animals, in the case of CLA and TVA isomers, in addition to diet, the spectrum of changes was also affected by the duration of the IPO application and diet x time interaction was found (*p* < 0.05, *p* < 0.01). The increase in the content of CLA isomers and TVA was probably related to the high supply of CLA isomer mixtures in the feed dose of cows and sheep (1% DM) and their transformations. According to Salsinha et al. [62], Bauman et al. [54], and Shingfield and Wallace [63], most dietary CLA isomers are subject to microbiological biohydrogenation in the rumen: first to *trans*-11 C18:1 (TVA) (from isomer C18:2 *cis*-9,*trans*-11) and *trans*-10 C18:1 (from isomer C18:2 *trans*-10,*cis*-12), and then to stearic acid (C18:0). A part of rumenic acid (c9,t11 CLA, RA) which is not hydrogenated to TVA or C18:0 in the rumen is absorbed from the gastrointestinal tract and, together with blood, is transported into the mammary gland. However, the contribution of this pathway in RA synthesis is negligible [64]. The predominant source of RA in milk fat (about 78%) is endogenous synthesis in the mammary gland from TVA as the rumen origin substrate, with Δ^9^-desaturase as the key enzyme [65,66]. A similar increase in the concentration of CLA and TVA in milk fat, as a result of infusion or supplementation of CLA isomers or their mixture, has been observed in other studies on cows [19,43,44] and sheep [17,46,52].

The desaturase index (DI) represents a proxy for Δ^9^-desaturase in the mammary gland and is calculated from fatty acid pairs that represent the product–substrate for this enzyme [67]. Milk fat desaturase indices are often altered during diet-induced MFD and CLA causes a reduction in milk fat as well as a shift in the DI. Oliveira et al. [46] and Perfield II et al. [59] demonstrated decreases in the ratios of C14:1/C14, 16:1/C16, C18:1/18:0, and RA/TVA, as did Chandler et al. [68] for C14:1/C14, 16:1/C16, and C18:1/18:0 (*p* < 0.01) and Hussain et al. [52] for 16:1/C16 (*p* < 0.05) as a result of CLA administration. In turn, Dohme-Meier and Bee [44] noted a decrease in 18:1/18:0 and an increase in 16:1/C16:0 (*p* < 0.05). In our study, the addition of IPO decreased the ratio of oleic acid (C18:1) to stearic acid (C18:0) and rumenic acid (c9,t11 CLA) to *trans*-vaccenic acid (C18:1 t11) (*p* < 0.01, *p* < 0.05), and had no effect on C14:1/C14 and C16:1/C16 ratios in either species of animal.

Given the negative role of C12:0, C14:0, and C16:0 acids, Ulbricht and Southgate [32] proposed the atherogenic index (AI). Conclusions concerning fat quality from the point of view of human diet may be drawn based on AI values. In our study, the AI value decreased from 10.6% to 20.5%, depending on the species of animal and the duration of the IPO additive administration.

## 5. Conclusions

The supplementation of the rations of dairy cows and ewes with isomerized poppy seed oil (IPO) reduced the content of milk fat, which had been confirmed in earlier studies, showing that dietary CLA lowered milk fat content in lactating animals. The decreased milk fat secretion was accompanied by a lower proportion of short-chain FAs (C4–10) as well as medium-chain FAs (C12–16) (i.e., laurynic, myristic, and palmitic acids) exhibiting atherogenic and thrombogenic effects, which is, in turn, consistent with the inhibitory effect of *trans*-10,*cis*-12 CLA on de novo FA synthesis. On the other hand, the content of biologically active FAs with pro-health properties (i.e., CLA isomers c9,t11 and t10,c12 and TVA) increased significantly in milk fat in both animal species as a result of IPO supplementation. In addition, it was observed that for sheep, the time of IPO supplementation had a greater effect on the spectrum of changes in individual fatty acids than in cows. This study demonstrated that isomerized poppy seed oil with high concentrations of CLA could be used in dairy animals to change the nutritional quality and associated health value of milk, which is beneficial from a human point of view.

## Figures and Tables

**Table 1:** Content of main fatty acids in poppy seed oil.

Fatty Acids (%)	Poppy Seed Oil	
	Raw Form	After Process of Alkaline Isomerization
C16:0	9.8	10.3
C18:0	2.1	3.9
C18:1 *cis*-9	12.3	12.8
C18:2 *cis*-9,*cis*-12	72.8	6.8
C18:2 *cis*-9,*trans*-11 (CLA)	nd ^1^	31.2
C18:2 *trans*-10,*cis*-12 (CLA)	nd	27.6
C18:2 *cis*-11,*trans*-13 (CLA)	nd	4.1
C18:3 *cis*-9,*cis*-12,*cis*-15	0.7	0.3
C18:3 isomers (CLnA) ^2^	nd	0.2
Σ CLA	nd	62.9

^1^ nd, <0.01 or not detected; ^2^ CLnA – conjugated linolenic acid, Σ C18:3 *cis*-8,*trans*-10,*cis*-12; *cis*-9,*trans*-11,*cis*-13; and *cis*-9,*cis*-1,*trans*-13.

**Table 2:** Ingredients and nutritive value of cow diets.

Specification	Treatment ^1^	
	CTRL	EXP
Ingredient, kg/head/day		
Maize silage	32.2	32.2
Silage from grass	10.3	10.3
Brewers’ spent grain	3.4	3.4
Wet beet pulp pressed	7.6	7.6
Complete mixture	4.5	4.5
Humokarbowit ^2^	1	1
Isomerized poppy seed oil	-	0.22
Mineral-vitamin mix ^3^	0.15	0.15
Nutritive value of ration		
DM (kg)	22.3	22.5
JPM ^4^	18.3	18.7
BTJN (g) ^5^	1858	1858
BTJE(g) ^6^	1856	1856
Ca (g)	135	135
P (g)	45	45

^1^ Treatments: CTRL, control group; EXP, experimental group supplemented with isomerized poppy seed oil (IPO). ^2^ Humic-mineral preparation (PHW Tronina, Raków, Poland). ^3^ Fatromix Bo W 3:1 contains Ca, 150 g; P, 50 g; Na, 130 g; Mg, 50 g; Zn, 8000 mg; Cu, 1500 mg; Mn, 6000 mg; I, 120 mg; Se, 30 mg; Co, 20 mg; vitamin A, 1,200,000 IU; vitamin D, 16,000 IU; vitamin E, 5000 mg; vitamin B, 210 mg; and vitamin H, 80 mg (Fatro Polska Sp. z o.o., Kobierzyce, Poland). ^4^ Feed unit for lactation. ^5^ Protein digested in the intestine when rumen fermentable nitrogen is limited. ^6^ Protein digested in the intestine when rumen fermentable energy is limited.

**Table 3:** Ingredients and nutritive value of sheep diets.

Specification	Treatment ^1^	
	CTRL	EXP
Ingredient, kg/head/day		
Concentrate feed containing:		
Crushed barley	0.7	0.7
Oat bran	0.3	0.3
Pape post-extraction meal	0.15	0.15
Grass hay	1.45	1.45
Humokarbowit ^2^	0.1	0.1
Isomerized poppy seed oil	-	0.02
Mineral-vitamin mix ^3^	0.03	0.03
Nutritive value of ration		
DM (kg)	2.11	2.13
JPM ^4^	1.84	1.88
BTJN (g) ^5^	187	187
BTJE (g) ^6^	182	182

^1^ Treatments: CTRL, control group; EXP, experimental group supplemented with isomerized poppy seed oil (IPO). ^2^ Humic-mineral preparation (PHW Tronina, Raków, Poland). ^3^ Polfamix OK contains Ca, 200 g; P, 120 g; Na, 60 g; Mg, 65 g; Zn, 2.5 g; Fe, 0.5 g; Co, 0.015 g; Mn, 0.01 g; I, 0.005 g; Se, 0.003 g; vitamin A, 300,000 IU; vitamin D3, 30,000 IU; and vitamin E, 1500 IU (Trouw Nutrition Polska Sp. z o.o., Poland). ^4^ Feed unit for lactation. ^5^ Protein digested in the intestine when rumen fermentable nitrogen is limited. ^6^ Protein digested in the intestine when rumen fermentable energy is limited.

**Table:** (**a**)

Item	CTRL ^1^			EXP ^2^			SEM ^3^	Effect^4^		
	7 d	14 d	30 d	7 d	14 d	30 d		D	T	D x T
**Fat Content, g/100 g of Milk**	4.11 ^A^	3.98 ^AB^	4.06 ^ABa^	3.57 ^BCb^	3.31 ^C^	3.24 ^Ca^	0.238	**	*	**
Fatty acids, g/100 g of total FAs										
C4:0	3.76 ^A^	3.58 ^A^	3.66 ^A^	2.32 ^Ba^	2.06 ^Bb^	2.21 ^B^	0.076	**	*	**
C6:0	2.42 ^A^	2.37 ^A^	2.42 ^A^	1.94 ^B^	1.87 ^B^	1.98 ^B^	0.023	**	NS	*
C8:0	1.67 ^A^	1.56 ^A^	1.63 ^A^	1.12 ^B^	1.23 ^B^	1.19 ^B^	0.018	**	NS	NS
C10:0	3.39 ^A^	3.28 ^A^	3.35 ^A^	2.46 ^B^	1.98 ^C^	2.09 ^BC^	0.126	**	**	**
C12:0	3.26 ^ab^	3.34 ^Aa^	3.32 ^Aa^	3.02 ^b^	2.89 ^B^	2.77 ^Bc^	0.072	**	*	*
C14:0	9.30	9.36 ^ab^	9.50 ^a^	8.84	8.24 ^c^	8.41 ^bc^	0.312	*	*	NS
C14:1 c9	0.70	0.68	0.72	0.71	0.67	0.69	0.087	NS	NS	NS
C15:0	0.62	0.65	0.66	0.62	0.64	0.63	0.096	NS	NS	NS
C16:0	27.36 ^ab^	27.66 ^a^	27.12	26.26	25.82 ^bc^	25.66 ^c^	0.534	*	NS	*
C16:1 c9	1.30	1.32	1.26	1.26	1.24	1.27	0.132	NS	NS	NS
C17:0	0.46	0.48	0.45	0.46	0.47	0.50	0.066	NS	NS	NS
C18:0	12.50 ^Aa^	12.72 ^ABa^	12.47 ^Aa^	14.74 ^BCb^	15.77 ^C^	16.10 ^Ca^	0.711	**	*	*
C18:1 t9	0.33	0.28	0.31	0.36	0.32	0.34	0.042	NS	NS	NS
C18:1 t10	0.72	0.67	0.72	0.74	0.72	0.69	0.058	NS	NS	NS
C18:1 t11 (TVA)	1.20 ^A^	1.28^A^	1.23^A^	2.68 ^Ba^	3.38 ^Bb^	2.98 ^B^	0.289	**	*	**
C18:1 other isomers *trans*	0.36	0.38	0.34	0.38	0.37	0.36	0.027	NS	NS	NS
C18:1 (OA)	23.52	22.58	23.07	23.21	23.93	24.12	1.312	NS	NS	NS
C18:2 (LA)	3.22	3.40	3.23	3.61	3.63	3.51	0.203	NS	NS	NS
C18:2 c9,t11 (CLA)	0.54 ^A^	0.52 ^A^	0.49 ^A^	1.04 ^B^	1.13 ^B^	1.09 ^B^	0.139	**	NS	*
C18:2 t10,c12 (CLA)	0.03 ^A^	0.04 ^A^	0.03 ^A^	0.06 ^Ba^	0.07 ^Bb^	0.06 ^B^	0.014	**	*	**
C18:2 other isomers CLA ^5^	0.02 ^A^	0.02 ^A^	0.02 ^A^	0.04 ^B^	0.04 ^B^	0.05 ^B^	0.007	**	NS	*
C18:3 (ALA)	0.62	0.57	0.60	0.59	0.57	0.55	0.069	NS	NS	NS
C20:0	0.06	0.05	0.07	0.06	0.07	0.07	0.012	NS	NS	NS
Σ LC-PUFAs ^6^	0.10	0.12	0.11	0.11	0.10	0.11	0.024	NS	NS	NS
Σ Unidentified	2.54	3.09	3.22	3.37	2.79	2.57	0.461	NS	NS	NS

^a–c^ Means within a row with different superscripts differ (*p* < 0.05). ^A–C^ Means within a row with different superscripts differ (*p* < 0.01). ^1^ CTRL, control group fed with total mixed ration (TMR) containing no additional oil; ^2^ EXP, experimental group fed with basal diet (TMR) containing the addition of 220 g/d (1% of DM) of isomerized poppy seed oil (IPO); ^3^ SEM, standard error of the mean; ^4^ probability of significant effects due to diet (D), time (T), and their interaction (D × T); * *p* < 0.05; ** *p* < 0.01; NS, not significant; ^5^ Σ, C18:2 *trans*-9,*cis*-11; *trans*-11,*cis*-13 and *trans*-11,*trans*-13; ^6^ LC-PUFAs, long-chain polyunsaturated fatty acids (FAs > 20).

**Table:** (**b**)

Item	CTRL ^1^			EXP ^2^			SEM ^3^	Effect ^4^		
	7 d	14 d	30 d	7 d	14 d	30 d		D	T	D x T
**Group of FA**										
SFAs ^5^	64.80 ^a^	65.05 ^Aa^	64.65 ^a^	61.85 ^b^	61.04 ^Bb^	61.61	1.021	*	NS	NS
SCFAs ^6^	11.24 ^A^	10.79 ^A^	11.06 ^A^	7.84 ^Ba^	7.14 ^Bb^	7.47 ^B^	0.286	**	**	**
MCFAs ^7^	40.54 ^a^	41.01 ^Aa^	40.60 ^ab^	38.74	37.59 ^bc^	37.47 ^Bc^	0.398	*	NS	*
MUFAs ^8^	28.13	27.19 ^A^	27.65 ^a^	29.34	30.63 ^B^	30.45 ^a^	0.386	*	NS	NS
PUFAs ^9^	4.54 ^Aa^	4.68 ^a^	4,48 ^Aa^	5.45 ^Bb^	5.54 ^Bb^	5.37 ^b^	0.242	**	NS	*
Σ CLA ^10^	0.59 ^A^	0.58 ^A^	0.54 ^A^	1.14^B^	1.24 ^B^	1.20 ^B^	0.059	**	NS	*
DI ^11^										
C14:1/(C14:0 + C14:1)	0.070	0.068 ^a^	0.071	0.074	0.075 ^b^	0.076	0.006	*	NS	NS
C16:1/(C16:0 + C16:1)	0.045	0.045	0.044	0.045	0.046	0.047	0.003	NS	NS	NS
C18:1/(C18:0 + C18:1)	0.653 ^a^	0.640	0.649 ^a^	0.611	0.603 ^b^	0.600 ^b^	0.018	*	NS	NS
c9,t11 CLA/C18:1 t11 + c9,t11 CLA	0.310 ^A^	0.289 ^ABa^	0.285 ^ABa^	0.279 ^B^	0.250 ^Ca^	0.268 ^BCb^	0.011	**	*	*
AI ^12^	2.08^ab^	2.15 ^Aa^	2.13 ^Aa^	1.86 ^b^	1.71 ^B^	1.73 ^B^	0.052	**	NS	*

^a–c^ Means within a row with different superscripts differ (*p* < 0.05). ^A–C^ Means within a row with different superscripts differ (*p* < 0.01). ^1^ CTRL, control group fed with total mixed ration (TMR) containing no additional oil; ^2^ EXP, experimental group fed with basal diet (TMR) containing the addition of 220 g/d (1% of DM) of isomerized poppy seed oil (IPO); ^3^ SEM, standard error of the mean; ^4^ probability of significant effects due to diet (D), time (T), and their interaction (D × T); * *p* < 0.05, ** *p* < 0.01, NS, not significant; ^5^ SFAs, saturated fatty acids; ^6^ SCFAs, short-chain fatty acids (FAs with C4–10); ^7^ MCFAs, medium-chain fatty acids (FAs with C12–16:0); ^8^ MUFAs, monounsaturated fatty acids; ^9^ PUFAs, polyunsaturated fatty acids; ^10^ Σ, CLA *cis*-9,*trans*-11; *trans*-10,*cis*-12; *trans*-9,*cis*-11; *trans*-11,*cis*-13 and *trans*-11,*trans*-13; ^11^ DI, desaturase index represents the ratio of product and substrate for Δ^9^-desaturase (double bonds are in the *cis* orientation unless otherwise indicated); ^12^ AI, atherogenic index calculated from (C12:0 + (4 x C14:0) + C16:0)/(MUFAs + PUFAs).

**Table:** (**a**)

Item	CTRL ^1^			EXP ^2^			SEM ^3^	Effect ^4^		
	7 d	14 d	30 d	7 d	14 d	30 d		D	T	D x T
**Fat Content, g/100 g of Milk**	6.35 ^Aa^	6.40 ^Aa^	6.32 ^A^	5.62 ^b^	5.11 ^B^	4.78 ^Ba^	0.326	**	*	**
Fatty acids, g/100 g of total FAs										
C4:0	2.27 ^A^	2.34 ^A^	2.25 ^ABa^	1.92 ^Bb^	1.75 ^BC^	1.62 ^C^	0.067	**	**	**
C6:0	1.98 ^A^	2.12 ^A^	2.05 ^A^	1.65 ^Ba^	1.42 ^BCb^	1.28 ^C^	0.043	**	**	**
C8:0	2.84 ^Aa^	2.68 ^A^	2.43 ^Ab^	1.92 ^Ba^	1.80 ^Bb^	1.84 ^B^	0.102	**	*	*
C10:0	5.22 ^A^	5.31 ^A^	5.44 ^A^	3.17 ^B^	2.97 ^B^	2.35 ^C^	0.167	**	*	*
C12:0	3.72 ^A^	3.68 ^A^	3.75 ^A^	3.02 ^Ba^	2.59 ^Bb^	2.75 ^B^	0.136	**	*	NS
C14:0	9.02 ^A^	8.76 ^a^	8.78 ^a^	8.17 ^Bb^	8.26 ^b^	8.30 ^b^	0.184	**	NS	*
C14:1 c9	0.82	0.85	0.82	0.82	0.78	0.80	0.045	NS	NS	NS
C15:0	1.06	1.02	1.04	0.98	1.01	0.94	0.126	NS	NS	NS
C16:0	25.06 ^a^	24.75	24.82 ^a^	24.02	23.68 ^b^	23.51 ^b^	0.412	*	*	*
C16:1 c9	1.89 ^a^	1.82	1.78	1.66 ^b^	1.70	1.72	0.184	*	NS	NS
C17:0	0.59	0.62	0.58	0.60	0.62	0.65	0.078	NS	NS	NS
C18:0	10.26 ^A^	10.58 ^A^	10.40 ^A^	13.68 ^Ba^	13.72 ^B^	14.66 ^Bb^	0.434	**	*	*
C18:1 t9	0.22	0.20	0.23	0.20	0.24	0.22	0.031	NS	NS	NS
C18:1 t10	0.60 ^Aa^	0.58 ^Aa^	0.63	0.66 ^b^	0.69 ^b^	0.70 ^B^	0.069	**	NS	*
C18:1 t11 (TVA)	1.17 ^A^	1.24 ^A^	1.19 ^A^	2.87 ^Ba^	3.24 ^C^	3.56 ^BCb^	0.206	**	**	**
C18:1 other isomers	0.30	0.29	0.27	0.28	0.31	0.29	0.044	NS	NS	NS
C18:1 (OA)	25.35	25.42	25.26	25.78	26.16	25.90	1.021	NS	NS	NS
C18:2 (LA)	3.48 ^Aa^	3.26 ^Aa^	3.31 ^Aa^	3.83 ^b^	4.32 ^Ba^	3.98 ^B^	0.342	**	*	**
C18:2 c9,t11 (CLA)	0.59 ^Aa^	0.65 ^Ab^	0.63 ^A^	1.08 ^Ba^	1.25 ^B^	1.38 ^Bb^	0.114	**	**	**
C18:2 t10,c12 (CLA)	0.03 ^A^	0.03 ^A^	0.03 ^A^	0.08 ^BCa^	0.07 ^B^	0.11 ^Cb^	0.018	**	**	**
C18:2 other isomers CLA ^5^	0.02 ^A^	0.02 ^A^	0.02^A^	0.03	0.04 ^B^	0.04 ^B^	0.006	**	NS	*
C18:3 (ALA)	0.98	0.95	1.02	0.95	0.98	1.04	0.166	NS	NS	NS
C20:0	0.15	0.15	0.14	0.14	0.15	0.13	0.045	NS	NS	NS
Σ LC-PUFAs ^6^	0.17	0.15	0.14	0.14	0.16 ^a^	0.13^b^	0.026	NS	*	NS
Σ Unidentified	2.21^a^	2.54	2.99^b^	2.35	2.09^a^	2.10^a^	0.348	NS	*	NS

^a–b^ Means within a row with different superscripts differ (*p* < 0.05). ^A–C^ Means within a row with different superscripts differ (*p* < 0.01). ^1^ CTRL, control group fed with complex mixture and grass hay (42%:58% of DM) containing no additional oil; ^2^ EXP, experimental group fed with basal diet containing the addition of 20 g/d (1% of DM) of isomerized poppy seed oil (IPO); ^3^ SEM, standard error of the mean; ^4^ probability of significant effects due to diet (D), time (T), and their interaction (D × T); * *p* < 0.05; ** *p* < 0.01; NS, not significant; ^5^ Σ, C18:2 *trans*-9,*cis*-11; *trans*-11,*cis*-13 and *trans*-11,*trans*-13; ^6^ LC-PUFAs, long-chain polyunsaturated fatty acids (FAs > 20).

**Table:** (**b**)

Item	CTRL ^1^			EXP ^2^			SEM ^3^	Effect ^4^		
	7 d	14 d	30 d	7 d	14 d	30 d		D	T	D x T
**Group of FA**										
SFAs ^5^	62.17 ^a^	62.01 ^a^	61.68	59.27	57.97 ^b^	58.03 ^b^	0.674	*	NS	NS
SCFAs ^6^	12.31 ^A^	12.45 ^A^	12.17 ^A^	8.66 ^Ba^	7.94 ^BCb^	7.09 ^Ca^	0.276	**	**	**
MCFAs ^7^	37.80 ^a^	37.19	37.35 ^a^	35.21 ^b^	34.53	34.56 ^b^	0.356	*	NS	NS
MUFAs ^8^	30.35 ^Aa^	30.40 ^Aa^	30.18 ^Aa^	32.27 ^b^	33.12 ^B^	33.19 ^B^	0.451	**	NS	*
PUFAs ^9^	5.27 ^A^	5.06 ^A^	5.15 ^A^	6.11 ^Ba^	6.82 ^Bb^	6.68 ^B^	0.262	**	*	*
Σ CLA ^10^	0.64 ^Aa^	0.70 ^Ab^	0.68 ^A^	1.19 ^Ba^	1.36 ^BCb^	1.53 ^C^	0.076	**	**	**
DI ^11^										
C14:1/(C14:0 + C14:1)	0.083	0.088	0.085	0.091	0.086	0.088	0.006	NS	NS	NS
C16:1/(C16:0 + C16:1)	0.07	0.068	0.072	0.065	0.067	0.068	0.005	NS	NS	NS
C18:1/(C18:0 + C18:1)	0.712 ^A^	0.706 ^A^	0.708 ^A^	0.663 ^Ba^	0.656 ^B^	0.638 ^Bb^	0.018	**	*	*
C18:2 c9,t11/ C18:1 t11 + C18:2 c9,t11	0.335 ^A^	0.344 ^A^	0.346 ^A^	0.273 ^B^	0.278 ^B^	0.279 ^B^	0.017	**	NS	*
AI ^12^	1.85 ^Aa^	1.82 ^Aa^	1.83 ^Aa^	1.56 ^Ba^	1.47 ^B^	1.47 ^Bb^	0.048	**	*	*

^a–b^ Means within a row with different superscripts differ (*p* < 0.05). ^A–C^ Means within a row with different superscripts differ (*p* < 0.01). ^1^ CTRL, control group fed with complex mixture and grass hay (42%:58% of DM) containing no additional oil; ^2^ EXP, experimental group fed with basal diet containing the addition of 20 g/d (1% of DM) of isomerized poppy seed oil (IPO); ^3^ SEM, standard error of the mean; ^4^ probability of significant effects due to diet (D), time (T), and their interaction (D × T); * *p* < 0.05; ** *p* < 0.01; NS, not significant; ^5^ SFAs, saturated fatty acids; ^6^ SCFAs, short-chain fatty acids (FAs with C4–10); ^7^ MCFAs, medium-chain fatty acids (FAs with C12–16:0); ^8^ MUFAs, monounsaturated fatty acids; ^9^ PUFAs, polyunsaturated fatty acids; ^10^ Σ, CLA *cis*-9,*trans*-11; *trans*-10,*cis*-12; *trans*-9,*cis*-11; *trans*-11,*cis*-13 and *trans*-11,*trans*-13; ^11^ DI, desaturase index represents the ratio of product and substrate for Δ^9^-desaturase (double bonds are in the *cis* orientation unless otherwise indicated); ^12^ AI, atherogenic index calculated from (C12:0 + (4 x C14:0) + C16:0)/(MUFAs + PUFAs).
